# Mechanisms Underlying Brain Aging Under Normal and Pathological Conditions

**DOI:** 10.1007/s12264-022-00969-9

**Published:** 2022-11-27

**Authors:** Menglong Jin, Shi-Qing Cai

**Affiliations:** 1 Institute of Neuroscience and State Key Laboratory of Neuroscience, CAS Center for Excellence in Brain Science and Intelligence Technology, Chinese Academy of Sciences, Shanghai, 200031 China; 2 University of Chinese Academy of Sciences, Beijing, 100049 China

**Keywords:** Healthy aging, Pathological aging, Cognitive impairment

## Abstract

Aging is a major risk factor for many human diseases, including cognitive impairment, which affects a large population of the elderly. In the past few decades, our understanding of the molecular and cellular mechanisms underlying the changes associated with aging and age-related diseases has expanded greatly, shedding light on the potential role of these changes in cognitive impairment. In this article, we review recent advances in understanding of the mechanisms underlying brain aging under normal and pathological conditions, compare their similarities and differences, discuss the causative and adaptive mechanisms of brain aging, and finally attempt to find some rules to guide us on how to promote healthy aging and prevent age-related diseases.

## Introduction

As we age, our bodies undergo many changes [1–3] that can be physiological and pathological. Just like other organs, the brain is susceptible to aging and it undergoes a series of structural and functional changes during aging [2, 4]. In the context of brain biology aging is a major risk factor for developing cognitive impairment like Alzheimer’s disease (AD), the most common dementia that affects ~55 million people worldwide and 15 million in China [5]. Thus, the development of new drugs or treatments to prevent or reverse cognitive impairment requires a comprehensive understanding of the biological events associated with the aging process in the brain.

In the past decades, our understanding of the molecular mechanism underlying the modulation of aging has been greatly expanded. Most remarkably, several signaling pathways have been identified as key aging modulators in *Caenorhabditis elegans* (*C. elegans*), flies, and mammals, suggesting that the biology of aging is conserved across species and the rate of aging is plastic [6, 7]. Nine common age-related molecular and cellular alterations have been proposed as hallmarks of aging [3]. Most of these hallmarks occur in aging neurons, except for cellular senescence and telomere attrition (both of which usually occur in proliferative peripheral tissues), which remain to be established [8]. A myriad of evidence has implied that these hallmarks also occur in pathological aging brains at a more severe level [9]. However, although brain atrophy and cognitive decline appear both in normal and pathological aging brains, the mechanisms underlying these phenomena are not exactly the same. Cognitive decline in normal aging is attributed to the disruption of circuits and synapses, without significant neuron loss, and this is in contrast to AD and related dementias, which are associated with extensive neuronal death [10, 11].

**Fig. 1:**
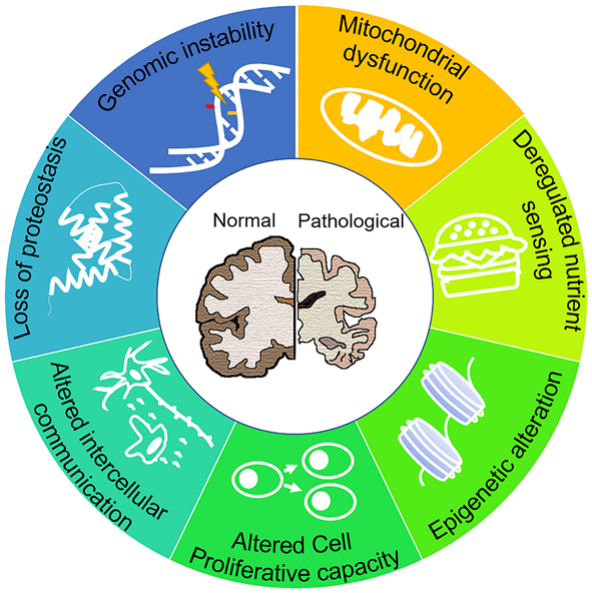
Molecular and cellular events occur in aging brains under normal and pathological conditions. Molecular and cellular alterations in genomic stability, mitochondrial function, nutrient sensing, epigenetic modification, cell proliferative capacity, intercellular communication, and proteostasis can be similar or different in aging brains under normal and pathological conditions.

In this review, we mainly focus on several molecular and cellular events (genomic instability, epigenetic alteration, mitochondrial dysfunction, deregulated nutrient sensing, altered cell proliferative capacity, altered intercellular communication, and loss of proteostasis; Fig. 1) to review the mechanisms of normal aging and how those processes interface with pathological aging of the brain.

## Genomic Instability

One common feature of aging is the accumulation of genetic damage throughout life [12]. Genomic DNA is regularly damaged by radiation, reactive oxygen species (ROS), DNA replication errors, and other exogenous or endogenous threats, and damaged DNAs are progressively accumulated during the normal course of aging. Such accumulation is more serious in aging neurons because excitatory synaptic activity produces more ROS, causing more DNA damage. Furthermore, neurons are post-mitotic cells and can not repair double-strand breaks by the more accurate homologous recombination pathway. Indeed, analyses of tissue samples from human and rodent brains have revealed that the amount of damaged nuclear and mitochondrial DNA increases and the capacity for DNA repair decreases during aging [8]. In the cortex of the aging human brain, promoter regions of a considerable proportion of genes are damaged, resulting in reduction of expression of a subset of genes associated with synaptic plasticity, mitochondrial function, and neuronal survival [13]. Interestingly, a similar pattern of DNA damage in vulnerable genes has also been found in *C. elegans*, *Drosophila*, and mouse [14–17], suggesting that selective DNA damage to gene promoter sequences is a conserved mechanism underlying age-related cognitive impairment.

Compared with the moderate DNA damage occurring in normal aging brains, the damage to genomic DNA in aging brains under pathological conditions is much more severe. Studies have shown that in human brains with AD, the genomic DNA is evidently disrupted, and the extensive DNA damage may eventually lead to the death of a large number of neurons [18]. Aggregated β-amyloid peptide (Aβ), a major pathological hallmark of AD, worsens the DNA damage by inducing aberrant synaptic activity, which elicits more ROS [19]. Similarly, enhanced oxidative DNA lesions and damage to mitochondrial DNA have been reported in the neurons of amyotrophic lateral sclerosis (ALS) and Parkinson disease (PD) patients, respectively [20–22].

Genomic instability is a key cause of age-related cognitive impairment. Accumulating evidence has demonstrated that impairment of DNA repair is sufficient to accelerate aging phenotypes and induce neurodegeneration [23]. Central dogma states that DNA transfers genetic information to RNA and produces functional proteins. Thus, DNA damage eventually affects the function of proteins, and therefore accumulation of DNA damage is a likely major cause of chronic systemic failures, which are further worsened under pathological conditions. This inevitably confers insults on the aging brain, consequently impairing cognitive function, and may drive the onset or progression of neurodegenerative diseases.

## Mitochondrial Dysfunction

Mitochondrial impairment has been regarded as one of the hallmarks of aging [3]. Neurons are more susceptible to mitochondrial impairment as they need high energy to be functional. In the aging brain, mitochondria exhibit a myriad of structural and functional changes, including morphological enlargement or fragmentation, membrane depolarization, an impaired electron transport chain, and increased oxidative damage to mitochondrial DNA (mtDNA) [3, 24]. The molecular mechanism underlying age-related dysfunction of mitochondria remains controversial. It has been suggested that mitochondrial dysfunction is due to the gradual accumulation of somatic mtDNA mutations. However, this hypothesis has been challenged by the fact that a cell has multiple mitochondrial genomes, which can tolerate the coexistence of wild-type and some mutant genomes [25]. Clearly, dysfunction of the mitochondrion is at least partially attributable to the age-related decline in the expression of mitochondrial proteins [13]. A recent study provides evidence that epigenetic changes contribute to mitochondrial dysfunction in the aging brain. The age-related upregulation of bromodomain adjacent to zinc finger domain 2B (BAZ2B) and euchromatic histone lysine methyltransferase 1 (EHMT1) underlie the down-regulation of genes involving core metabolism (mainly ribosome and oxidative phosphorylation), which have been consistently reported in patients with AD and mild cognitive impairment [26].

Pathological aging brains show more severe dysfunction of mitochondria. Many lines of evidence have suggested that mitochondrial dysfunction plays a central role in the onset and development of neurodegenerative diseases. The age-related reductions in oxidative phosphorylation and accumulation of mtDNA damage are aggravated in the brains of AD patients [27–29]. Impaired mitochondrial biogenesis and fission–fusion balance, as well as decreased mitophagy, have also been reported in AD patients’ brains [30, 31]. When it comes to the relationship between mitochondria and pathological brain aging, PD should not be ignored [32, 33]. Although the exact cause of the disease is not clear, aberrant mitochondrial clearance by autophagy is a major factor in the death of dopaminergic neurons. The protein kinase PTEN-induced putative kinase 1 (PINK1) accumulates on the outer membrane of the mitochondria, and then phosphorylates and activates the E3 ligase activity of Parkin, which ubiquitinates outer mitochondrial membrane proteins to trigger selective autophagy [34]. Healthy neurons effectively remove damaged mitochondria through mitochondrial phagocytosis; while in PD neurons, excessive mitochondrial damage causes a failure in the clearance of damaged mitochondria, resulting in extensive neuronal death.

Mitochondria establish and maintain the aging rate of the entire organism [24], but the effect of mitochondrial damage on the aging phenotypes is not simply linear. Previously, it was supposed that an energetic crisis in aging brains caused by accumulating damaged mitochondria accelerates functional deterioration and triggers neurodegenerative diseases. However, recent genetic and pharmacological evidence has discovered that, although severe mitochondrial dysfunction may cause cell death, mild respiratory defects extend the lifespan. Down-regulating the function of the electron transport chain in neurons has been reported to extend the lifespan of *C. elegans* [24]. On the other hand, several reports have demonstrated that caloric restriction, genetic/pharmacological restoration of nicotinamide adenine dinucleotide (NAD^+^), or manipulation of a glia-neuron signal can increase mitochondrial function and activation of the mitochondrial unfolded protein response (UPR^mt^) to mitigate many of the detrimental effects of aging and/or prolong the healthspan [35–39]. Perhaps maintaining mitochondrial function at the proper level, not too low or too high, is most beneficial for promoting healthy aging.

## Deregulated Nutrient Sensing

**Fig. 2:**
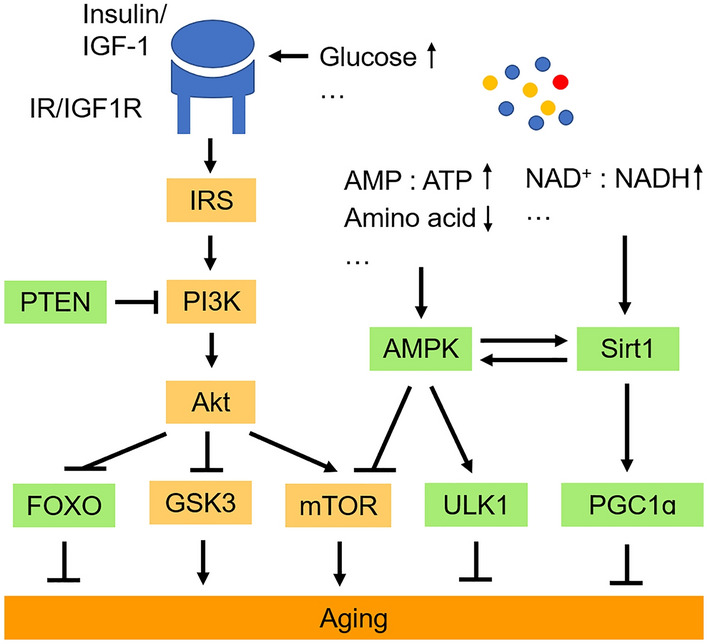
Nutrient-sensing pathways and aging. Molecules proposed to favor aging and prevent aging are shown in orange and green, respectively. IR, insulin receptor; IGF-1, insulin-like growth factor 1; IGF1R, IGF-1 receptor; IRS, IR substrates; PI3K, phosphoinositide 3-kinase; Akt, AKT serine/threonine kinase; FOXO, forkhead box O; GSK3, glycogen synthase kinase-3; mTOR, mammalian target of rapamycin; AMPK, adenosine 5′ monophosphate-activated protein kinase; ULK1, Unc-51 like autophagy activating kinase 1; Sirt1, sirtuin 1; PGC1ɑ, PPARG coactivator 1 alpha; AMP, adenosine monophosphate; ATP, adenosine triphosphate; NAD^+^, nicotinamide adenine dinucleotide; NADH, nicotinamide adenine dinucleotide hydride.

The function of the nutrient-sensing system changes with age. Previous studies have shown that several nutrient-sensing pathways play a role in regulating longevity and aging [40]. As shown in Fig. 2, these pathways include insulin and the insulin-like growth factor 1 (IGF-1) signaling (IIS) pathway, the mammalian target of rapamycin (mTOR) pathway, the adenosine 5′ monophosphate-activated protein kinase (AMPK) pathway, and the sirtuin signaling pathway.

The IIS pathway, which represents nutrient abundance and anabolism, is the most conserved aging-modifying pathway in the process of evolution, and its downstream targets, forkhead box O (FOXO) family transcription factors, are also highly conserved in regulating longevity across species: inhibiting the activity of the IIS pathway extends the lifespan of yeast, worms, flies, and mammals [40]. Brain IIS plays a key role in sensing and regulating body metabolism, as well as regulating synaptic plasticity, learning, and neural development [41]. It is worth noting that, although the IIS pathway plays a small role in glucose metabolism in the brain, it is essential for maintaining a healthy brain metabolism [42]. The IIS pathway sends signals of the body's energy status to the brain and regulates eating behavior: excessive insulin inhibits eating, while a decreased insulin level causes overeating through the IIS pathway in the hypothalamus [43, 44]. Interestingly, growth hormone and IGF-1 levels decrease during normal aging, and this phenomenon has also been reported in mouse models of premature aging [45]. One possible explanation for this is that cells compensate for aging and age-related declines by downregulating the function of IIS pathways. mTOR kinase, another highly-conserved factor for sensing and responding to nutrient signals (including signals related to the availability of amino-acids), also plays a key role in modulating aging, and administration of the mTOR inhibitor rapamycin is considered to be one of the most effective approaches to increasing the lifespan in mammals [46, 47]. Of note, the activity of the mTOR signaling pathway also decreases during normal brain aging [48], but in AD patients, the mTOR pathway is hyperactivated [49]. Some other pathways (like AMPK and sirtuins) sense nutrient scarcity and catabolism, and they act in an opposite way to modulate aging. Studies have shown that the responsiveness of AMPK signaling decreases with age and enhancing AMPK activity delays aging [50]. In general, the available evidence has demonstrated that anabolic signaling accelerates the rate of aging and reduced nutritional signaling increases longevity [51].

In aging brains, reduced glucose metabolism is found in many brain regions, particularly marked in the temporal and parietal lobes, and the motor cortex [52]. This alteration is much more profound in these regions of elderly patients with dementia [53]. In patients with neurodegenerative diseases, such as AD and PD, reduced brain glucose consumption and energy production are associated with altered IIS, mTOR, and AMPK signaling. Studies have shown that brain insulin resistance accelerates aging, while maintaining brain insulin sensitivity delays aging [54]. An abnormal distribution and cellular localization of insulin receptors (IR and IGF1R) have been reported in AD neurons, suggesting possible insulin resistance in the AD brain [55]. The dysfunction of insulin and IGF-1 signaling may also contribute to the progress of AD, as insulin signaling plays a critical role in the clearance of Aβ and inhibiting Tau aggregation [56]. Disruption of insulin receptor substrates 2 (IRS2) promotes Tau phosphorylation by activating glycogen synthase kinase-3β in the *Irs2*^−/−^ mouse brain [57]. Interestingly, other lines of evidence support the idea that inhibition of IIS alleviates AD-related phenotypes [58]. Neuron-specific knockout of IRS2 (but not IR or IGF1R) delays Aβ accumulation in a mouse model of AD [59], suggesting that the role of IIS in AD pathology is distinct in different cell types. Inconsistent changes in the AMPK pathway have also been reported in the AD brain. Generally, AMPK activity decreases in the AD brain, but it is abnormally activated in neurons bearing neurofibrillary pre-tangles and tangles; activated AMPK accumulates near Aβ plaques and tau tangles instead of in the nucleus, where AMPK usually functions [60]. Thus, moderating the sensitivity of AMPK pathways rather than simply inhibiting or activating them (which may cause resistance) may be more effective for relieving AD symptoms [54].

The manipulation of nutritional signaling pathways may provide effective approaches to alleviate brain aging and prevent age-related neurodegenerative diseases. Dietary restriction is probably the best known way to extend the lifespan and the healthspan [61–63]. A reduced inflammatory response and improved capacity of DNA repair are found in dietary-restricted mice and rats, as a result of upregulation of genes associated with inhibition of the inflammatory response and DNA synthesis/repair [64–66]. In animal models of AD and stroke, dietary restriction protects neurons in the brain from degeneration [67]; however, no benefit of dietary restriction on alleviation of the disease has been reported in an animal model of ALS [68]. Recently, studies on intermittent fasting (IF) are gaining increasing interest [69]. Studies suggest that IF has no positive short-term effects on cognition in healthy subjects, but has benefits in relieving the symptoms of epilepsy, AD, and multiple sclerosis [70]. Although the exact mechanism of IF in healthy aging is still unclear, the process is thought to be associated with the IIS pathway, the mTOR pathway, and the like [71].

## Epigenetic Alteration

Epigenetics is the best choice for organisms to adapt to a changeable environment: heritable changes in gene expression occur while the DNA sequence does not change. Epigenetic changes include alterations in DNA methylation patterns, post-translational modification of histones, chromatin remodeling, and expression of non-coding RNAs [72]. Generally, diversity of modification helps individuals adapt to their environment; however, studies have shown that as individuals age, heterogeneity of DNA modifications in the brain decreases at both the individual and tissue levels, and convergence of neighboring DNA modifications suggests that there exist some rules of age-related alteration in DNA modifications during brain aging [73]. Interestingly, changes in the levels of DNA methylation at 353 CpG sites have been proposed as an epigenetic clock (known as DNA methylation age) to predict the biological age of human tissues including the brain [74]. Generally, histone methylation levels in the promoter regions of many synaptic function-related genes increase, while histone acetylation levels decrease in aging brains [75]. These epigenetic changes ultimately affect gene expression, leading to cognitive decline during aging.

Epigenetic alterations have also been widely reported in pathological brain aging [76], and these alterations may be different from those that occur in normal aging. For example, the methylation level in the promotor region of many AD risk genes (such as APP, PS1, and BACE1) changes significantly in AD patients’ brains compared with that of age-matched normally aging brains [77]. However, the global changes in DNA methylation under AD conditions is controversial: a decreased DNA methylation level has been found in the cell model of AD, but an increased DNA methylation level has been reported in a mouse model of AD [78]. Normal aging results in significant accumulation, while AD entails a marked loss, of H4K16ac in the proximity of genes associated with the aging process and AD, suggesting that the marker H4K16ac plays a distinct role in normal aging and age-associated neurodegeneration [79].

On the other hand, some epigenetic changes could be a continuous regulatory mechanism for normal and pathological brain aging. Studies have reported an increase in the levels of H3K9me2 and H3K9me3 in aging brains of aged mice, mouse models of AD and Huntington’s disease (HD), as well as AD and HD patients [80, 81]. Pharmacological inhibition of the histone methyl transferases SUV39H1 and EHMT1/EHMT2 decreases the levels of H3K9me3 in the hippocampus and improves cognitive function in aged mice and a mouse model of AD, respectively [82, 83]. Strikingly, a recent study found that the expression levels of EHMT1 and an epigenetic reader BAZ2B increase with age, and are positively correlated with the progress of AD in the prefrontal cortex of human brains. Ablation of Baz2b enhances mitochondrial function in the brain and improves cognitive function in aged mice by affecting the H3K9 methylation level in the promotor regions of genes associated with core metabolism [26].

MicroRNAs (miRNAs) provide another epigenetic mechanism for regulating brain aging. Although dysfunction of miRNAs affects neuroinflammation, cognitive decline, and other age-related deteriorations, currently little is known about functional changes in brain miRNAs during aging [84]. Microarray data indicate that some miRNAs, including miR-139-5p, miR-342-3p, and miR-204, exhibit increased expression levels in the hippocampus of aged mice, and they may suppress the surface expression of neurotransmitter receptors and contribute to age-related cognitive decline [85, 86]. Recent evidence suggests that miRNAs can be packed into exosomes, which can be transported cross tissues and probably coordinate the ageing rate of different organs. Astrocytic apolipoprotein E3 (APOE3) vesicles have been reported to carry diverse regulatory miRNAs, which specifically silence genes involved in neuronal cholesterol biosynthesis, and ultimately promote histone acetylation and activate gene expression in neurons. In contrast, APOE4, a risk factor for AD, carries fewer miRNAs and thus down-regulates the acetylation level of histone in the promoter regions of synapse-related genes, ultimately leading to repressed expression of these genes and a decline in cognitive ability [87].

Overall, the potential reversibility of epigenetic alterations provides exciting chances to alter the trajectory of brain aging under both normal and pathological conditions. Studies have shown that some epigenetic factors regulate healthy aging, such as BAZ2B and EHMT1, and they may be targets for slowing brain aging and treating age-related diseases [26]. Expression of octamer-binding transcription factor 3/4, SRY-Box transcription factor 2 (Sox2), Kruppel-like factor 4, and the proto-oncogene c-Myc (OSKM, also known as Yamanaka factors) in cells reshapes epigenetic markers and reprograms cells to a pluripotent state, as well as erasing cellular markers of senescence [88–90]. Periodic expression of OSKM for a short period of time improves the aging phenotypes of aged mice and prolongs the life span of progeroid mice [90], and the effect on delaying aging increases with the cumulative time of partial reprogramming in aged mice, suggesting that epigenetic changes occur early during aging and that earlier interventions may get better results [91]. Notably, despite controversy, reprogramming glial cells into neurons is an exciting and challenging approach to treating neurodegenerative diseases [92, 93]. However, more work remains to be done to understand epigenetic regulation in aging brains under both normal and pathological conditions.

## Altered Cell Proliferative Capacity

In most organs, one of the most obvious age-related hallmarks is the decline in the regenerative potential of tissues, which is a result of telomere attrition, stem cell exhaustion, and cellular senescence [94, 95]. Evidence shows that telomere alteration affects the rate of aging. It has been demonstrated that mice with shortened and lengthened telomeres show decreased and increased lifespan, respectively. Pathological dysfunction of telomeres accelerates aging, while experimental stimulation of telomerase delays aging [96, 97]. However, neurons are permanently postmitotic and neuronal telomeres do not shorten in aging brains. Interestingly, changes in the telomere have shown an opposite role in different studies of pathological brain aging. Telomere lengths are shorter in AD patients, and telomerase-deficient mice showed signs of neurodegeneration [98, 99]. However, another study in the APP23 transgenic mouse have shown that, although telomere shortening disrupts adult neurogenesis and the maintenance of neurons after mitosis, it reduces the formation of amyloid plaques and alleviates the deficits in learning and memory [100].

In the adult brain, neurogenesis only occurs in a few regions, including the hippocampal dentate gyrus, sub-ventricular zone (SVZ), and olfactory bulb [101, 102]. Age-related changes in neurogenesis have been found in both normal and pathological aging brains. During normal and pathological aging, a decrease of neurogenesis in the hippocampus, SVZ, and olfactory bulb may lead to cognitive and olfactory deficits [103]. Neural stem cell transplantation has been considered as a potential treatment for some age-related neurodegenerative diseases associated with neuronal loss [104]. However, aberrant neurogenesis has been reported in aging brains with neurodegenerative disorders, including AD, PD, and HD [105]. For example, the human HD brain exhibits greater neural progenitor cell (NPC) proliferation that is proportional to the severity of the disease, and modifies the pathology of the disease [106]. Both increased and decreased neurogenesis have been reported in the brains of AD model mice and AD patients [105, 107]. Some studies on postmortem brain tissue from AD patients found a higher level of neurogenesis in the hippocampus [108], while other studies found that neurogenic markers like Musashi-1, Sox2, and doublecortin are downregulated in the brains of AD patients [109, 110]. This discrepancy may be explained by difference in the animal model used and the stage of the disease. Overall, the capacity for neurogenesis may change differently in normal and pathological brain aging, despite the fact that their states are both abnormal and may contribute to the cognitive impairments.

NPCs and proliferation-competent glial cells can undergo senescence. Cells with features of senescence have been found in aging brains under both normal and pathological conditions [111]. Senescence alters cellular morphology and proteostasis, decreases autophagy-lysosome function, and enhances the secretory activity of cells, which leads to a pro-inflammatory senescence-associated secretory phenotype [112]. Senescent cells accumulate during aging and negatively influence a heathy lifespan. In AD model mice, the telomeres in microglia shorten during continuous replication, leading to replicative senescence and contributing to AD pathology [113]. Although neurons no longer undergo mitosis, they show some features of senescence in aging brains; the level of senescence-associated beta-galactosidase activity increases in neurons in the CA3 region of aged rat brains, and a DNA damage-induced senescence-like phenotype has also been reported in aging neurons [114, 115]. Removal of senescent cells in the brain by pharmacological intervention, chimeric antigen receptor T cell therapy, or genetic/antibody-mediated depletion alleviates the inflammation and cognitive impairment in naturally aging mice, and prevents tau-dependent pathology and cognitive decline in mouse models of neurodegenerative diseases [116–119].

## Altered Intercellular Communication

Maintaining connections with other cells is critical for preserving the functions of neurons. With increasing age, the communication between neuronal cells changes significantly, causing decreased synaptic connections, increased inflammation, and degenerated neurovascular units (NVUs) [3, 8].

The fidelity of neural networks within and between brain regions is disturbed with age. Functional imaging studies have revealed a global loss of integrative function in aging human brains. Age-related dysfunction of neural networks is not due to a loss of neurons (which is minimal in normal aging brains), but to abnormal synaptic connections that form the basis of neural circuits in the hippocampus and prefrontal cortex [120]. The expression levels of most synaptic function-related genes are down-regulated [13] and the integrity of the white matter (which is composed of bundles of axons) is compromised during normal aging of the human brain [121]. Not surprisingly, these alterations may render neurons more vulnerable to degeneration under pathological conditions [122], reinforcing the importance of maintaining synaptic function to achieve healthy aging of the brain. In support of this notion, recent studies have demonstrated that improving synaptic function by enhancing neurotransmitter levels alleviates the age-related deterioration of cognitive function in old people and animal models [123, 124]. However, other lines of evidence have shown that inhibition of neuronal excitability delays aging. Mutations that cause defects in specific sensory neurons extends the lifespan in *C. elegans*, and ablation of RE1 silencing transcription factor causes abnormal neural excitation in the aged mouse brain and accelerates aging through the IIS pathway [125, 126]. These findings suggest that higher neural activity is not always better, and the changes of neural connections may also be an adaptive mechanism in response to aging.

In addition to synaptic connections, factors such as neurotrophic and inflammation factors from other cells influence neuronal survival. In neurodegenerative diseases, synaptic inputs may be altered by inhibiting anterograde axonal transport and/or axonal degeneration, resulting in reduced release of transmitters and neurotrophic peptides [127]. During aging, glial cells, particularly microglia, often exhibit an activated state, causing long-term and chronic inflammation in the nervous system [128], which may lead to cognitive impairments as evidenced by the findings that in both normal and pathological brain aging, inhibition of the inflammatory responses has been reported to improve learning and cognition [129, 130]. For example, activating the cholinergic anti-inflammatory pathway, inhibiting the activation of microglia, or restoring the metabolic levels of myeloid cells in aging mice alleviates inflammation in the nervous system and restores cognitive function [131–133].

NVUs also degenerate with age [134]. Changes of NVUs in aging usually result in impaired integrity of the blood-brain barrier (BBB) and infiltration of blood-derived proteins, affecting the blood oxygen supply to nerve cells. The degeneration of NVUs eventually impairs cognitive function and increases the risk of stroke and cerebral small-vessel disease, among others [135, 136]. Interestingly, unique changes in NVUs occur in some neurodegenerative diseases. For example, Aβ42 has been reported to disturb the organization of junctional complexes containing tight and adherent junctions between endothelial cells, causing leakage of plasma protein and more severe damage in brains with AD [137]. The deposition of α-synuclein and matrix metallopeptidase 3 damages the BBB and exacerbates inflammation in PD patients’ brains [138–140]. Thus, it is reasonable to assume that protecting the functions of NVUs is a promising strategy for preventing some neurodegenerative diseases.

## Loss of Proteostasis

The proteostasis network regulates cellular protein homeostasis and it consists of the protein biosynthetic machinery, the molecular chaperone system, and protein clearance pathways including the proteasome and autophagy systems. The loss of proteostasis, a failure of aging cells in responding to proteotoxic challenges, has been considered a hallmark of organismal aging [3]. A recent study has proposed that age-related changes in translational efficiency initially drive the deterioration of proteostasis [141]. Genetic and pharmacological strategies to improve proteostasis promote healthy aging in animal models and delay the onset of age-related pathologies associated with protein aggregation [142].

In aged brains, the functions of protein chaperones and proteolytic systems decrease significantly [143]. Once the capacity of these systems decreases below a threshold level, some aggregation-prone proteins aggregate and exist in an insoluble state. The threshold is lowered in some stress conditions, such as the presence of disease-associated mutations in genes encoding aggregation-prone proteins [142]. Indeed, in age-related neurodegenerative diseases, loss of protein homeostasis often shows more severe pathological features. For example, aggregation of Aβ and hyperphosphorylation of Tau are the most obvious pathological features of AD; and a large amount of α-synuclein aggregates in dopaminergic neurons in PD patients [144]. Stress response pathways shape the proteostasis network, affecting the process of aging and the onset/progress of neurodegeneration. The aggregation of unfolded proteins perturbs the homeostasis in the endoplasmic reticulum (ER) and triggers ER stress-induced activation of the unfolded protein response (UPR), an initially adaptive mechanism that restores cellular proteostasis [145, 146]. However, studies have demonstrated that chronic activation of the UPR in the ER is associated with neuronal degeneration in post-mortem brain tissues from patients with neurodegenerative diseases [145, 146]. Thus, activation of the UPR in the ER has distinct and even opposite effects on the progress of neurodegeneration, probably depending on the stage of disease.

It is worth noting that the relation between cognitive impairment and toxic protein aggregation remains controversial. In about a quarter of patients diagnosed as having AD-like diseases, the level of Aβ plaques and tau tangles does not reach the diagnostic threshold of AD [147, 148]. Many old people who exhibit extensive Aβ plaque accumulation only have minimal neuronal loss and a marginal decline in cognitive function [149, 150]. These suggest that there may be neuroprotective mechanisms that we have not yet found that influence the role of proteostasis during brain aging.

## Conclusions and Perspective

**Fig. 3:**
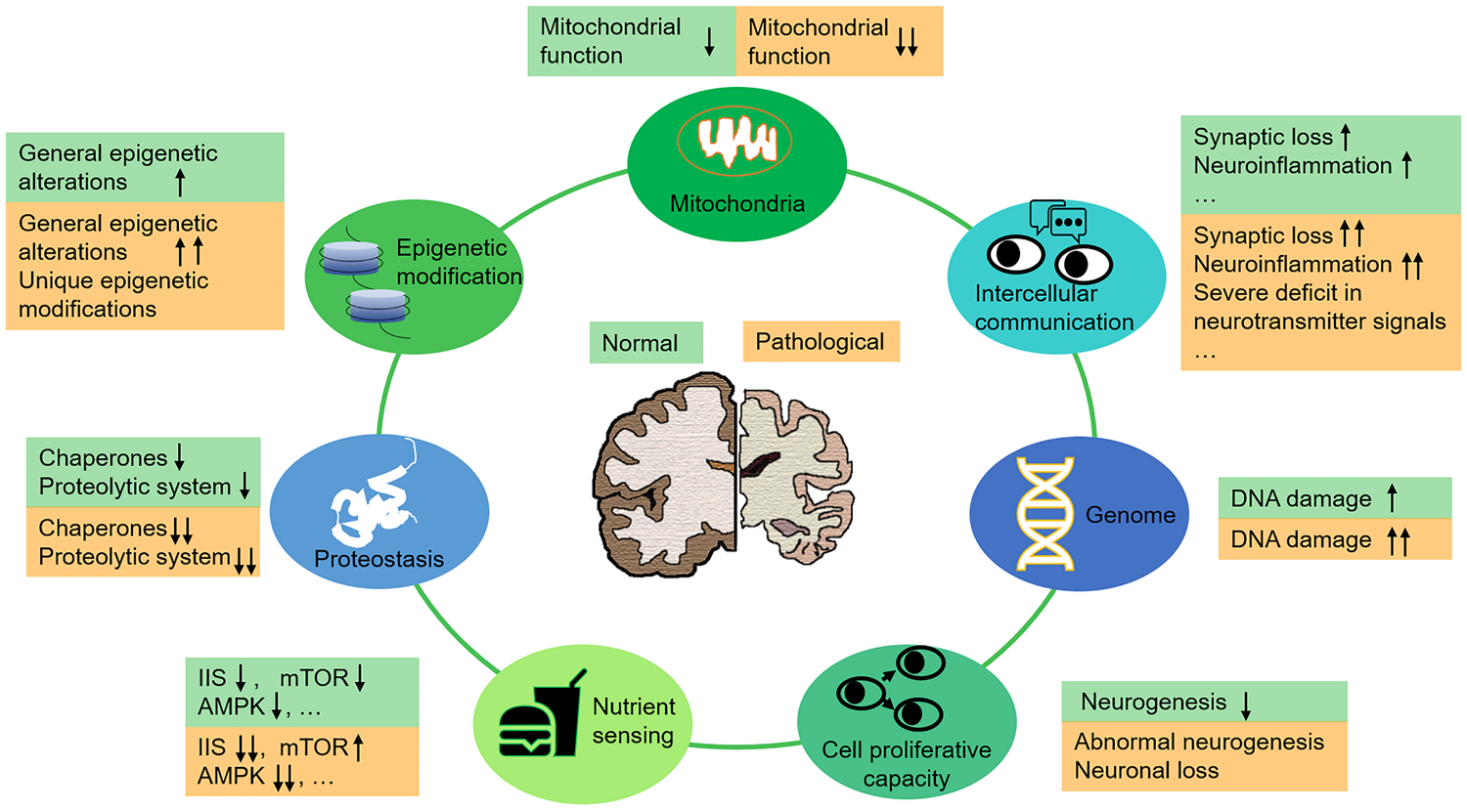
Similar and different molecular and cellular changes in aging brains under normal and pathological conditions. Changes occurring in normal and pathological aging brains are shown in green and in orange, respectively. These changes are only examples, not the full range of changes during normal and pathological brain aging. IIS, the insulin and IGF-1 signaling pathway; mTOR, mammalian target of rapamycin.

Brain aging is a major risk factor for many neurodegenerative diseases, but the mechanisms underlying the transition from normal aging to neurodegenerative disorders are complex and remain to be fully elucidated. The hallmarks of brain aging have been associated with the pathogenesis of neurodegenerative diseases, which are quite common among older populations, leading to the hypothesis that neurodegeneration is accelerated brain ageing [151]. Indeed, as shown in Fig. 3, some hallmarks of brain aging occur in both normal and pathological brain aging, but they are more exacerbated in a pathological state. These are genomic instability, deregulated nutrient sensing, loss of proteostasis, and mitochondrial dysfunction. However, some unique molecular and cellular processes occur in normal aging or neurodegenerative diseases. For instance, some changes in intercellular communication (such as NUV damage caused by Aβ in AD [137]) and epigenetic modifications (such as methylation level in the promotor region of many AD risk genes [77]) specifically occur in aging brains under pathological conditions. Strikingly, other molecular and cellular changes (such as alteration in telomeres and stem cells) in the brain are distinct between normal aging and neurodegeneration, showing an antagonistic mechanism. It is worth noting that the opposite changes that occur in neurodegenerative diseases ultimately exacerbate the pathological phenotype rather than alleviate the aging phenotype.

**Fig. 4:**
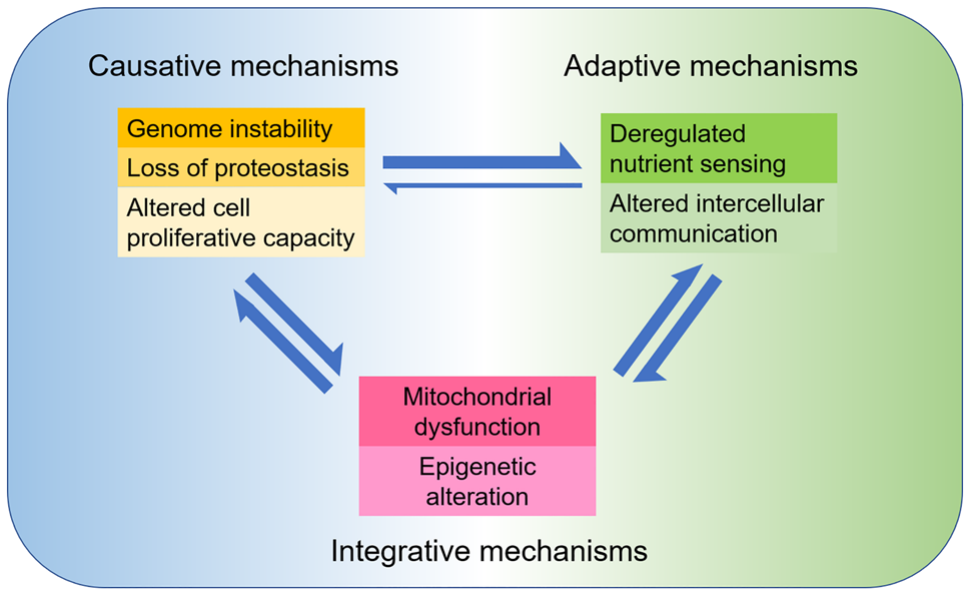
Functional interconnections between the molecular and cellular events occurring in aging brains under normal and pathological conditions. The seven molecular and cellular events occurring in aging brains can be divided into three categories. Genome instability, loss of proteostasis, and altered cell proliferative capacity are supposed to be causative mechanisms; deregulated nutrient sensing and altered intercellular communication are mostly adaptive mechanisms; while mitochondrial dysfunction and epigenetic alteration could be both causative and adaptive for brain aging and neurodegeneration.

Brain aging is a key regulator of longevity and functional deterioration. Molecular and cellular alterations associated with the aging process in the brain could be adaptive factors for maintaining neural function or causative factors for deteriorating systemic functions [3]. Generally speaking, as shown in Fig. 4, genome instability, loss of proteostasis, and altered cell proliferative capacity are causative mechanisms for cognitive impairment [23, 95, 142]; while deregulated nutrient sensing and altered intercellular communication are mainly considered to be adaptive mechanisms for brain aging because down-regulation of nutrient sensing and neuronal excitability have been reported to extend lifespan [45, 125]. Mitochondrial dysfunction and epigenetic changes could be both causative and adaptive mechanisms for brain aging [24, 25, 72]. For example, mild downregulation of mitochondrial function promotes longevity, while severe impairment of mitochondria leads to neuronal death and neurodegeneration. Yet, the causative and adaptive mechanisms are not absolutely inconvertible; the adaptive molecular and cellular changes in the aging brain may also contribute to the decline of some subsets of behaviors. Future comprehensive studies on single-cell transcriptomics [152] of normal, protected, and pathological aging human brains may help to further clarify the causative and adaptive age-related molecular and cellular events in a cell type-specific pattern.

It is worth noting that the hallmarks of brain aging are interrelated and interdependent, and none of them occur in isolation. For example, age-related accumulation of DNA damage and epigenetic aberrations alters gene expression, resulting in dysfunction of mitochondria, deregulation of nutrient sensing, and loss of proteostasis; all of which in turn lead to more severe cytotoxicity and genomic instability, finally causing pathological changes in intercellular communication and transforming physiological changes into pathological changes in the whole brain. Accumulating evidence has demonstrated that caloric restriction protects aging brains from deterioration by stimulating mitochondrial biogenesis and neural signaling pathways in neurons [8], supporting a link between nutrient sensing and mitochondrial function in modulating aging of the brain.

Given that normal and pathological brain aging are closely linked, approaches that promote healthy aging are worth trying to treat aging-related diseases. Recent studies have demonstrated that exposure of an aged animal to young blood improves the aging phenotypes, and plasma replacement has also been reported to be beneficial for treating AD [153–155]. A combination of removal of senescent cells with transient somatic reprogramming synergistically extends lifespan and improves health [156]. On the other hand, brain aging is a complex and integral process, therefore future development of therapeutic approaches for treating neurodegenerative diseases needs a greater understanding of not only the distinct molecular changes occurring in pathological aging brains, but also the biological context of normal aging of the brain. Many mouse models of AD develop disease symptoms too early, which may not be suitable for the search for the true pathogenesis of the disease [157, 158]. Researchers must take these questions into account when using these model mice.
